# A Comparative Electromyographic Analysis of Flying Squirrel and 3-Point Quadripod Exercise for Lumbar Multifidus Muscle Activations among Healthy Female Subjects

**DOI:** 10.3390/healthcare11060833

**Published:** 2023-03-12

**Authors:** Qais Gasibat, Babina Rani, Denis Čaušević, Wajida Perveen, Cristina Ioana Alexe, Alina Elena Albina, Dan Iulian Alexe

**Affiliations:** 1 Department of Sports Studies, Universiti Putra Malaysia UPM, Selangor 43400, Malaysia; 2 Department of Physical Rehabilitation & Medicine (Physiotherapy), Post Graduate Institute of Medical Education and Research, Chandigarh 160012, India; 3 Faculty of Sport and Physical Education, University of Sarajevo, 71000 Sarajevo, Bosnia and Herzegovina; 4 School of Allied Health Sciences, CMH Lahore Medical College & IOD (NUMS Rawalpindi), Lahore 54810, Pakistan; 5 Department of Physical Education and Sports Performance, Faculty of Movement, Sports and Health Sciences, “Vasile Alecsandri” University of Bacău, 600115 Bacău, Romania; 6 Department of Theory and Methodology of Motor Activities, Faculty of Physical Education and Sports, University of Craiova, Alexandru Ioan Cuza nr.13, 200585 Craiova, Romania; 7 Department of Physical and Occupational Therapy, Faculty of Movement, Sports and Health Sciences, “Vasile Alecsandri” University of Bacău, 600115 Bacău, Romania

**Keywords:** electromyography, core stability, low back pain, back muscles, exercises

## Abstract

Physical therapists employ several exercises to alleviate low back pain (LBP). Electromyography (EMG) examination of exercises can monitor muscle activation to help clinicians determine the exercise’s effect on stabilisation, endurance, or strength. This study evaluated surface EMG activity comparison for Flying Squirrel Exercise (FSE) and the novel 3-Point Quadripod Exercise (3-PQE) to find the most effective exercise for stimulating the lumbar multifidus (LM) muscle. The study recruited 64 healthy young females (19–24 years). Raw data were normalized and are expressed as the percentage of maximum voluntary isometric contraction (%MVIC). The test–retest reliability of the EMG recordings was estimated using intraclass correlation coefficient (ICC3,1). One-way ANOVA was used to statistically analyse and compare the EMG amplitudes during the two exercises. The ICCs for 3-PQE and FSE were 0.94 (SEM, 21.7% MVIC) and 0.87 (SEM, 19.05% MVIC), respectively. The 3-PQE (69 ± 26% MVIC) demonstrated significantly higher activity than did FSE (30 ± 18% MVIC) (F = 15.573, *p* = 0.001). Thus, 3-PQE might be a feasible strategy for the prevention and rehabilitation of LBP in females.

## 1. Introduction

The spinal response to sudden loads is greatly influenced by the stability of the lumbar spine. When trunk load magnitude increases, lumbar stability increases in proportion to a rise in trunk muscle activation [1]. The failure of active (muscle) or passive (osseo-ligamentous) structures to provide stability can therefore result in low back pain (LBP). Corrupted signals from injured mechanoreceptors, resulting from subfailure injuries of spinal ligaments and disc, may cause chronic LBP due to muscle control dysfunction [2]. As pointed out by the Global Burden of Disease Study 2019, the global prevalence rate for LBP was higher for females [3]. Reproductive and hormonal variables, such as a hysterectomy and delayed or irregular menstrual cycle, are associated with a lower incidence of LBP in males than in females [4]. It is worth noting that the efficacy of an intervention programme must be determined in a healthy population first before its results can be applied to a clinical set of patients [5,6].

Choosing an appropriate exercise may be advantageous when the right and relevant muscles are targeted [7,8]. Muscle activation in the process of mobility might prove effective in the management of patients with LBP, as well as in the prevention of future occurrences of LBP in healthy individuals [9]. Although exercises designed to increase muscular activation have gained recognition in the rehabilitation of LBP patients, there are insufficient and ambiguous data regarding the effectiveness of this method [10].

Of late, the multifidus muscle in the lumbar region has been the subject of much researcher interest. The lumbar multifidus (LM) muscle is the most critical muscle for lumbar segment stability based on the association between its atrophy and LBP recurrences [10,11,12,13]. In LBP subjects, various abnormal features of LM have been documented in the literature. These include motor control deficits of altered recruitment pattern [14], histological changes with replacement of LM muscle tissue with fatty infiltrates [15], and muscle atrophy [16]. Its return to normal size in individuals who participated in an exercise programme was accompanied with the stress and degeneration of the LM muscle tissue, resulting in reduced function [17,18]. Macedo et al. [19] discovered that atrophy of the LM muscle persisted for at least 10 weeks in acute LBP subjects who did not adhere to the exercise programme, but patients who participated in the stabilisation exercise programme that emphasised isolated LM and deep abdominal muscle contractions regained the normal muscle size [19].

The multifidus cross-sectional area can be an indicator of deconditioning or hypotrophy of the muscle and suggest abnormality. Cross-sectional area measurement is reliably performed with musculoskeletal ultrasound imaging via established protocols [20]. The results of these investigations have shown that, barring highly athletic populations, those who have low back pain tend to have smaller multifidus cross-sectional areas [20,21]. The multifidus cross-sectional area is correlated with pain, indicating that a smaller multifidus may increase the likelihood of experiencing pain [22]. In those with acute nonspecific low back pain, bilateral LM atrophy and asymmetry have been observed [23].

Evidence proving the superiority of one rehabilitation exercise programme over another for patients with LBP is not available in the literature. As a result, a vast array of activities has been applied for the progressive strengthening of muscles affected in LBP. Electromyography (EMG) is the gold standard measurement tool for examining the muscle’s activity and therefore allows for the analysis of back muscle activation levels during training, which can assist a therapist in selecting the most appropriate exercises. Kavcic et al. [24] have published the most extensive data of the EMG activity of back muscles during a range of activities involving the lower back, but other investigators have focused on EMG studies during some selected set of particular exercises [24,25,26,27,28,29,30,31,32]. Certain exercises have not yet been investigated using EMG analysis in females. It is therefore imperative to determine the most effective muscular activation exercise in females. The current study’s aim is to investigate the EMG activity of the lumbar multifidus during the Flying Squirrel Exercise (FSE) and compare it with the novel 3-Point Quadripod Exercise (3-PQE) in healthy female subjects. The authors hypothesise a higher electromyographic activity for LM muscle during 3-PQE relative to FSE. This comparison will serve as evidence for application of these exercise programmes in the prevention and rehabilitation of LBP and/or lumbar radiculopathy.

## 2. Materials and Methods

### 2.1. Participants

Sample size was determined using G*Power (version 3.1.6); the alpha level and effect size were set at 0.05 and 0.23, respectively [33]. For the study, 64 young females between the ages of 19 and 24 years, with a BMI between 18.5 and 24.9 kg/m^2^, were recruited. Subjects were ineligible to participate in the study if any of the following conditions were met: (1) any history of a leg injury; (2) infection or prior surgery in the back; (3) back pain; (4) experience with trunk muscle strength testing; (5) exercising more than twice a week or doing it competitively; (6) being treated for any musculoskeletal, systemic, or neurological illness; (7) pregnancy/lactation; or (8) a BMI higher than 35 kg/m^2^. Volunteers for this study had a mean age ± SD of 21.48 ± 1.50 years (Table 1). Volunteers for this study were recruited from the Sport Studies Department at University Putra Malaysia UPM in Serdang, Selangor, Malaysia. All participants provided their written consent by signing an informed consent form. All the protocols, procedures, and tools used in this study had prior approval from the Research Ethics Board of the Ethical Committee for Research Involving Human Subjects at University Putra Malaysia (JKEUPM; 099).

### 2.2. Procedures

**Figure 1:**
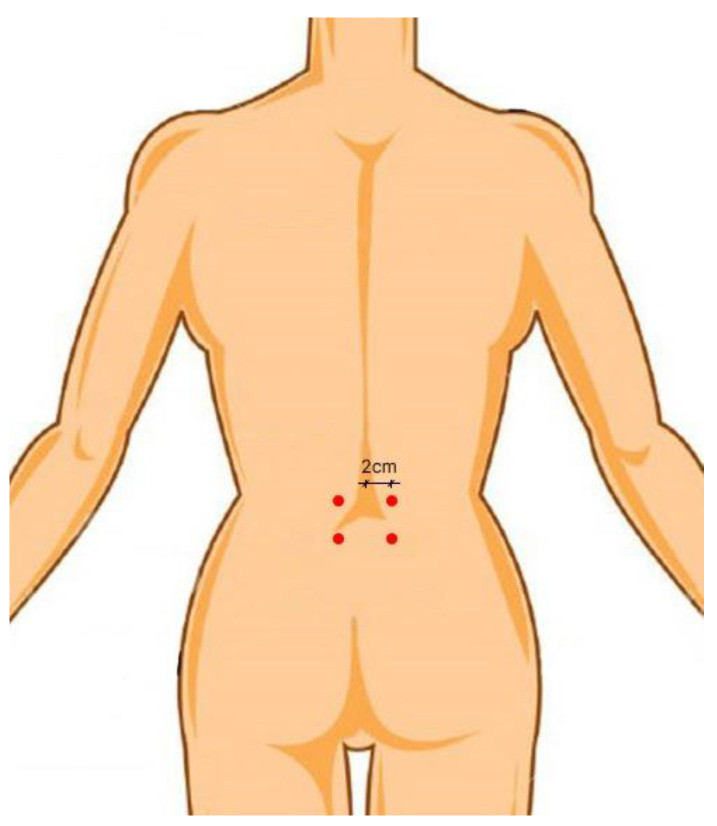
Schematic diagram for electrode placement for the lumbar multifidus muscle.

Each participant received training in the necessary muscle tests and exercises prior to having electrodes placed. After making sure that the subjects would successfully complete each intervention protocol, we prepared the intended electrode placement sites on skin with fine sandpaper and sterilised it with 70% isopropyl alcohol. If there was an overabundance of hair, it was shaved off. When required, the researchers made sure to cue the subjects to avoid any unwanted or trick movements during the test session exercises. The Nihon Kohden II machine (Nihon-Kohden Co., Tokyo, Japan) was used for EMG data collection at 200–500 microvolt and 10 milliseconds with a low- and high-cut filter at 50 Hz and 3 kHz, respectively. In order to record the electromyographic activity, we used disposable silver/silver chloride electrodes (Noraxon USA, Inc., Scottsdale, AZ, USA). In addition, electrode placement was secured using micropore tape to prevent the electrodes from slipping around and to maintain their adherence while the subjects were working out. Bilateral LM muscles were sampled for EMG recordings in all the subjects. The LM muscle was studied by means of a 2-channel EMG. Each electrode was positioned 2 cm laterally, with the lumbosacral junction as the target. The anterior superior iliac spine served as a reference point for the electrode. The electrodes were placed in accordance with the guidelines established in a prior study [34]. The electrode placement was performed as depicted in the schematic diagram in Figure 1. A maximum voluntary isometric contraction (MVIC) test was conducted for LM muscles using prone trunk extension, with additional resistance provided at the upper thoracic region. The amplitude of the resulting EMG signal was recorded for use in data normalization. Manual muscle testing typically used by physical therapists served as a guide for the test positions employed, albeit additional manual resistance was provided to the back muscles in the tests performed in the experiment [7,9]. The highest amount of force that could be incorporated manually was applied and kept constant for 5 s. Verifying proper electrode placement during manual muscle tests also involved analysing the EMG amplitudes recorded.

The activities completed are detailed in Table 2. Each set of exercises was completed in random order and held isometrically for 5 s. The MVIC test and each test exercise were performed three times, with a 30 s rest interval in between and a 5 min rest between the change of exercise. The average of the three readings was then used for analysis. The MVIC test was performed after completion of the two test exercises. EMG signal amplitudes during manual muscle tests helped verify the electrode placement. All the processes carried out for EMG analysis were in accordance with similar previous studies [29,32].

### 2.3. Statistical Analysis

The data analysis was carried out using IBM SPSS software 22.0 for Windows (SPSS Inc., Chicago, IL, USA). The same day test–retest reliability of the LM muscle EMG recordings was determined with the intraclass correlation coefficient (ICC3,1). The differences in LM activity for the two exercises was examined using one-way repeated-measures analysis of variance (ANOVA). The significance was established at the *p* < 0.05 level.

## 3. Results

### 3.1. Electromyography Activity

Table 3 summarizes the mean EMG activity (expressed as a percent of MVIC) of the LM muscle for each test exercise. A significantly greater EMG signal amplitude was observed for 3-PQE (mean ± SD: 69 ± 26% MVIC) relative to FSE (mean ± SD: 30 ± 18% MVIC) (F = 15.573, *p* = 0.001).

### 3.2. Reliability of EMG Recordings

The same day test–retest ICCs and standard error of mean (SEM) for the EMG recordings from the LM muscle during the 3-PQE and FSE were 0.94 (SEM, 21.7% MVIC) and 0.87 (SEM, 19.05% MVIC), respectively, indicating a good consistency in the EMG recordings.

## 4. Discussion

There is no proof in the literature that one exercise programme is superior to another; thus, physical therapists tend to utilise a variety of techniques while repairing the low back muscles of their patients. The objective of this study was to analyse the muscle activity of LM while performing FSE and 3-PQE in healthy female volunteers using surface EMG. The results demonstrated significantly higher activity during 3-PQE relative to FSE (F = 15.573, *p* < 0.05). In terms of boosting LM activations in the clinical scenario, our findings imply that the 3-PQE intervention is more effective than is the FSE intervention.

In 2001, Arokoski et al. [25] discovered a strong connection between the surface and intramuscular activity of the normalised EMG amplitude for LM muscle at the second and fifth lumbar levels. A study by Danneels et al. [35] reported the reliability of surface electrodes for the multifidus muscle. However, definitive conclusions regarding the usage of surface electrodes for LM requires further exploration. Despite the unavoidable existence of crosstalk between the two muscles, we believe this study offers significant insights into the activity of the lumbar multifidus muscles during different workouts. Regarding LM, multiple investigations have indicated decreased fatigue resistance, histological changes [36], and atrophy [35], as well as a diminished capacity to actively recruit muscle [37], in patients with chronic low back pain (CLBP). Consequently, the amplitude of the EMG signal can provide information on the amount of force produced [9], and the intensity of the exercise and can be employed for strengthening muscle and for the rehabilitation and prevention of LBP.

Shima et al. [38] compared the surface electromyography activity of LM and erector spinae muscle in young healthy male individuals during arm and leg ergometer exercises. They recruited a smaller sample (15 subjects) and used surface EMG and ECG as outcome measurement tools. The percentage MVIC values during the leg-ergometer exercise were 2.6 ± 2.1, 6.9 ± 5.7, and 10.3 ± 6.8%. The %MVIC values increased with increased workloads (50 W and 100 W conditions vs. rest condition, *p* < 0.01; 100 W condition vs. 50 W condition, *p* < 0.01, all). Meanwhile, in the arm-ergometer exercise, the percentage MVIC values of the multifidus were 4.6 ± 2.9, 9.2 ± 5.6, and 12.6 ± 7.6% at the rest, 50 W, and 100 W conditions, respectively. The difference in percentage MVIC values was significantly higher for the arm-ergometer exercise than for the leg-ergometer exercise at the rest condition (*p* < 0.05). However, there was no significant difference between the two types of exercises at the 50 W or 100 W conditions. There was also no significant difference in the range of increase in muscle activity of the multifidus between the leg- or arm-ergometer exercises. Both leg and arm exercises were found to be effective in training the target muscles; however, more activity was observed for the erector spinae during arm ergometer exercises. The reason for this may be the difference of the angle of pelvic tilt in the two positions. The skeletal mass among the study population may differ due to habitual physical activity, which appears to be a limitation of this study.

In Brazil, Gonçalvez et al. [39] studied the effects of manual therapy and photobiomodulation (PBM) on twenty subjects with low back pain. The two groups were randomly allocated to two intervention groups receiving the prescribed kinesio therapeutic protocol for two months consecutively. Along with the Oswestry disability index, surface EMG was applied for LM and transverse abdominal muscles. The position of the side bridge and the Sorensen test maintenance time were recorded in seconds. The activation of most of the evaluated muscles was decreased. The left multifidus and the left transversus abdominus muscles in the manual therapy group had a large effect size (g = 0.73 and g = −0.93, respectively), and the right multifidus of the PBM group obtained a moderate effect size. In terms of MVIC, both groups achieved large effect size and greater activation.

While previous research has looked at LM’s EMG activity on the back surface during various activities, a direct comparison with our findings is not possible due to methodological and experimental setup differences. Ekstrom et al. [29] employed surface EMG to analyse the activation of the LM and longissimus thoracis (LT) muscles during a range of common back rehabilitation exercises. Their results indicated that during the quadruped arm and lower limb lift, the EMG amplitude in the LM was 46 ± 21% MVIC. For the quadripod posture with a neutral spine, another study found that the EMG amplitude in the LM muscle varied from 29 ± 11% to 41 ± 12% of MVIC during right upper and left lower extremity lifts [9]. The EMG activity of the LM muscles during the quadripod exercise was found to be between 25% and 50% of the maximal voluntary isometric contraction in a comparable study [40]. Our investigation found similar results for FSE (30 ± 18% MVIC), while the 3-PQE demonstrated significantly greater activity (69 ± 26% MVIC). 3-PQE showed 2.3-fold higher activity levels compared to FSE.

Since LM activity has been shown to be higher in females than males, the fact that only females participated in our study may account for the heightened LM activations we observed [7]. Heightened lumbar lordotic curvature in females [41] may possibly be attributable to the greater activation of the LM muscle [32,42]. In a study of individuals with CLBP, the lumbar lordosis position in the quadripod leg raise and quadripod leg–arm raise exercises increased LM activity by 9.7 and 16.9% for the MVIC, respectively, as compared to the neutral posture [41]. Composite stabilisation training programmes have been studied before for their ability to promote lumbar lordosis and, by extension, multifidus activation in females [7]. A large gender gap was discovered in a prior analysis of the bridge and unilateral bridge exercises by Arokoski et al. [25]. On average, female’s LT and LM muscles had greater EMG signal amplitude values than those of males. The LM muscle activity was greatest during prone bilateral leg extension at 76.7 ± 25.1% MVIC for females and ranged from 29% to 35% MVIC for males to 53% to 57% MVIC for females during the bridge exercise. On a similar note, findings from this study indicated that the LM is equivalently activated during 3-PQE (69 ± 26% MVIC).

Patients with CLBP were examined by Ferreira et al. [43], who looked at the effects of three different rehabilitation programmes on pain and functional impairment: standard exercises, stabilisation exercises, and manual treatment. While short-term exercise routines caused more discomfort than they were worth, long-term stabilisation training was shown to reduce pain and impairment more effectively compared to any other technique (including usual medical care, conventional physiotherapy treatment, education, therapeutic modalities etc.) [44]. In a 2003 review by Ferreira et al., spinal manipulation was shown to be more effective than massage, diathermy, and no treatment at all for patients with nonspecific LBP persisting for less than three months [45]. In a similar manner, Cairns et al. [46] analysed the effects of physical therapy on the stability and mobility of females with LBP. It was determined from their data that both training methods are successful in lowering pain intensity among the groups throughout a 12-week intervention period. Furthermore, the motor control exercise (MCE), which includes training the pre-activation of deeper spinal muscles, was revealed to be superior to manual treatment for LBP, disability, and quality of life outcomes at intermediate follow-up in participants with chronic nonspecific LBP. Short-term follow-up showed that MCE was superior to other exercises in reducing the severity of the impairment [19].

During the quadripod exercise, other researchers have found erector spinae activity levels between 20% and 50%, and LM activity values between 27% and 56% [9,28,29]. Traditional strengthening exercises have also been shown to help people with LBP [46,47]. Two recent papers found that patients with recurrent LBP benefited either from general exercises for trunk muscular activation or from particular exercises for lumbar stabilisation, with the latter leading to greater functional improvement and less discomfort [46,47]. In comparison to the other exercises previously examined in the prone position, 3-PQE has the additional benefit of placing no or limited compressive spinal stress on the delicate lower lumbar and lumbosacral spine, which may be especially helpful for those who have experienced previous injuries in these areas. Further study is required to determine whether or not 3-PQE also contributes to the proprioceptive advantage of weight bearing.

To the best of our knowledge, our study is the first to compare the FSE and 3-PQE modalities electromyographically in healthy female participants. Based on our results and the related literature, it may be concluded that both the FSE and 3-PQE training modalities effectively produce higher muscle activations, the efficacy of which needs to be investigated through further interventional training programs. Furthermore, it may be concluded that low- to moderate-load exercises may be appropriate for increasing muscle activation, which may be partially explained by the known abundance of slow twitch fibres in the LM [48]. It would be interesting to investigate the effects of the novel 3-PQE in patients with low back and sacroiliac dysfunction.

### 4.1. Limitations of the Study

Because of time, logistic, and budgetary constraints, this study was not carried out on a broad scale. No other muscle was studied along with LM, so a comparison cannot be drawn for the relative muscle activities during the test exercises (FSE and 3-PQE). The study was also hampered by a number of technical challenges, such as the management of the subjects, the supply of appropriate laboratory equipment, and the increased competition for space in the laboratory brought on by the high demand from other students. The study must be further conducted on various populations (healthy, sports, clinical pathology) to confirm its strength benefits.

### 4.2. Strengths and Practical Implications of the Study

This study supports the superiority of the 3-PQE, finding it to be the more convenient and efficacious rehabilitation tool for LBP of relevant cause as compared to FSE. Additionally, the study has demonstrated that using EMG signals to identify muscle activation is beneficial since it allows researchers to precisely pinpoint the ideal intervention programme that can intensify the muscles’ activation in the lumbar region among healthy female volunteers. In the long run, this would be considerably beneficial to female patients with a history of LBP. Restoration of functional status in chronic LBP patients can be facilitated after obtaining proper therapy by employing an appropriate and simple intervention like 3-PQE. From biomedical perspectives, physiological sensors are the principal elements of biosignal processing and electronic medical rehabilitation techniques. The EMG biosensors denote the connection between biomedical signals and rehabilitation. Therefore, the application of this technology has enabled the identification of appropriate intervention programmes that could benefit physiotherapists in adopting the rehabilitation of female participants diagnosed with LBP.

Since this study was conducted on healthy volunteers, it is prudent to exercise caution in projecting these findings to a clinical scenario.

## 5. Conclusions

The outcomes of this study demonstrate that LM was activated during FSE and 3-PQE, with results ranging from 21% to 96% MVIC. The 3-PQE programme (69 ± 26% MVIC) proved to be more effective in activating the LM than did the FSE (30 ± 18% MVIC). 3-PQE warrants further research to prove its effectiveness for the purpose of general fitness or LBP prevention and rehabilitation,

The study’s findings may be useful to therapists leading a patient through an LBP rehabilitation programme. Because there is such a significant difference in muscle activation in participants during any given activity, the programme must still be tailored to the patient’s impression of exercise intensity.

## Figures and Tables

**Figure 2:**
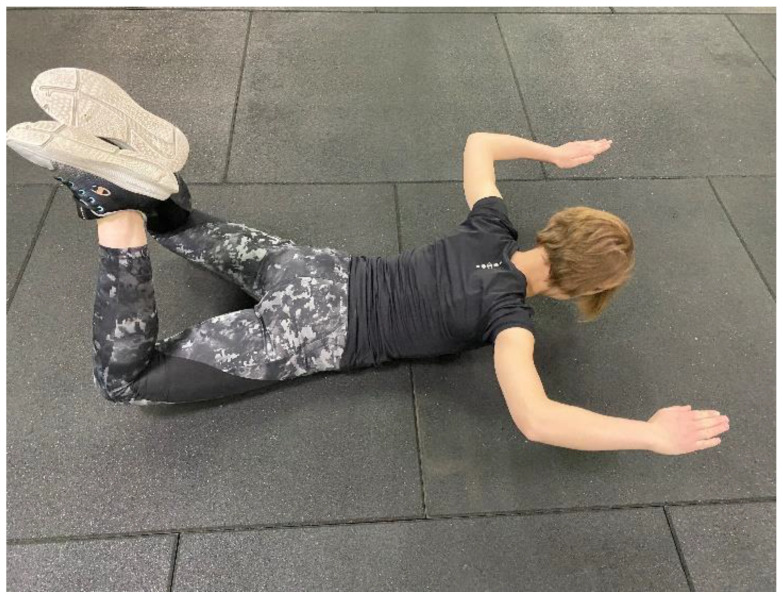
Flying Squirrel Exercise (FSE).

**Figure 3:**
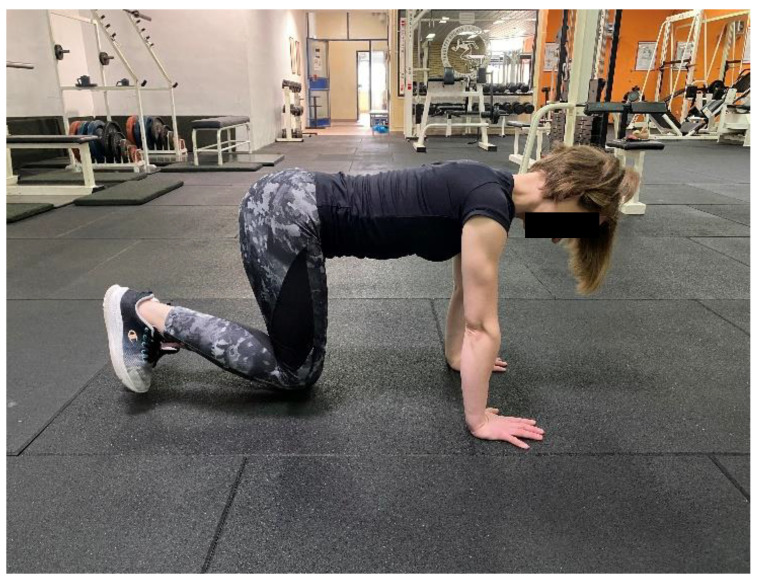
3-Point Quadripod Exercise (3-PQE).

**Table 1:** Baseline data of subjects (*n* = 64).

	Mean	SD
Age (years)	21.48	1.50
Height (cm)	152.86	13.7
Weight (kg)	50.65	7.43
BMI (kg/m^2^)	20.47	1.31

**Table 2:** Procedures of the selected exercises.

Name of Exercise	Muscle	Procedure
**FSE (Figure 2)**	LM	Subject is asked to lift the legs off the ground while lying prone with the knees contracted and the hips internally rotated. With shoulders in external rotation, subject is asked to lift the arms off the ground while flexing the elbows. The position is maintained for the next five seconds.
**3-PQE (Figure 3)**	LM	In the Quadripod position, subject is asked to engage the core and then lift one knee off the couch by just an inch. The subject holds this position for 5 s while maintaining a neutral spine.

FSE: Flying Squirrel Exercise, 3-PQE: 3-Point Quadripod Exercise, LM: lumbar multifidus.

**Table 3:** EMG activity of the LM muscle during FSE and 3-PQE (*n* = 64).

EMG Activity (%MVIC)				
Exercise	Test (Mean ± SD)	Retest (Mean ± SD)	ICC	95% CI for ICC
FSE	30 ± 18	31 ± 03	0.87	0.84–0.89
3-PQE	69 ± 26	68 ± 67	0.94	0.91–0.96

## Data Availability

The data presented in this study are available upon request from the corresponding author. The data are not publicly available due to privacy restrictions.
